# Mental state and emotion detection from musically stimulated EEG

**DOI:** 10.1186/s40708-018-0092-z

**Published:** 2018-11-29

**Authors:** Avinash L. Tandle, Manjusha S. Joshi, Ambrish S. Dharmadhikari, Suyog V. Jaiswal

**Affiliations:** 1 NMIMS University, Mumbai-56, India; 2 Mpower The Foundation, Mumbai, India; 3 H.B.T. Medical College and Dr. R.N. Cooper Mun. Gen. Hospital, Mumbai, India

**Keywords:** EEG, Music, Emotion, Machine learning

## Abstract

This literature survey attempts to clarify different approaches considered to study the impact of the musical stimulus on the human brain using EEG Modality. Glancing at the field through various aspects of such studies specifically an experimental protocol, the EEG machine, number of channels investigated, feature extracted, categories of emotions, the brain area, the brainwaves, statistical tests, machine learning algorithms used for classification and validation of the developed model. This article comments on how these different approaches have particular weaknesses and strengths. Ultimately, this review concludes a suitable method to study the impact of the musical stimulus on brain and implications of such kind of studies.

## Introduction

The human brain is a spectacularly complex organ, how the brain processes an emotion having very little acquaintance. Discovering how the brain processes the emotion will impact not only in artificial emotional intelligence, human–computer interface but also have many clinical implications of diagnosing affective diseases and neurological disorders. There are several multidisciplinary and collaborative researches across the globe happening using different modalities of brain research and to investigate how the brain processes emotion. There are many ways to evoke the emotion; music is the excellent thriller and elicitor of emotion [1]. During listening unique music the physiological responses of subjects like shivering, speeding heart, goosebumps, laughter, lump in throat, sensual arousal and sweating [2]. Tuning in to the music incorporates different mental means, for example, observation, multimodal combination, focus, reviewing memory, syntactic handling and the preparing of significant data, activity, feeling and social discernment [3]. Thus, music is a potent stimulus for evoking the emotions and investigating processing functions of the human brain. There are several modalities of brain research categorised depending on how it measures neuronal activity of the brain, direct imaging, and indirect imaging, direct imaging measures electrical or magnetic signal generated due neuronal activity directly, e.g. EEG (electroencephalogram) and MEG (magnetoencephalogram), whereas indirect imaging fMRI (functional magnetic resonance imaging), PET (positron emission tomography), etc., measure neuronal activity using oxygen consumption of neurone. Indirect measuring has an excellent spatial resolution in case of PET around 4 mm and f MRI 2 mm but low temporal resolution low for PET 1–2 min and fMRI 4–5 s [4] and other enlisted disadvantages

- Subject has to take radionuclide dye
- Claustrophobic
- Noisy
- Mostly used for clinical research purpose
- Highly expensive machine cost ($20,00,000–800,000) and scanning cost ($800–1500.) [4]

Direct imaging reasonable good spatial resolution and excellent temporal resolution 1 ms in case of EEG its 10 mm but having several advantages to carry the stimulus-based experiment [4]

- Non-ionising
- Simple to work, portable
- Silent
- No claustrophobia
- Comparatively Inexpensive Machine cost ($1000–$10,000) and Scan cost ($100) [4]
- Simple to plan incitement test
- Easy to configuration/assemble HCI (human–computer interface) research and applications

This article reviews the literature of clinical and engineering domain to quantify the impact of music stimulus. The aspect of items of evaluations among literature is

- Type of population and sample
- EEG recording environment and recording Machine
- Stimulus Type, duration of the stimulus, emotion Model
- Feature extraction transform, feature extracted
- Brainwave investigated
- Statistical test and machine learning algorithm used
- Assessment of model

The paper is written using an approach of a summary of reviews, an analysis of surveying aspects and synthesis of reviewing aspects and organised in sections as follows: Sect. 2 covers the structural information of brain, Sect. 3 represents literature selection and analysis, Sect. 4 shows summary of review, and Sects. 5, 6 and 7 represent discussion, suggested approach and conclusion, respectively

**Fig. 1:**
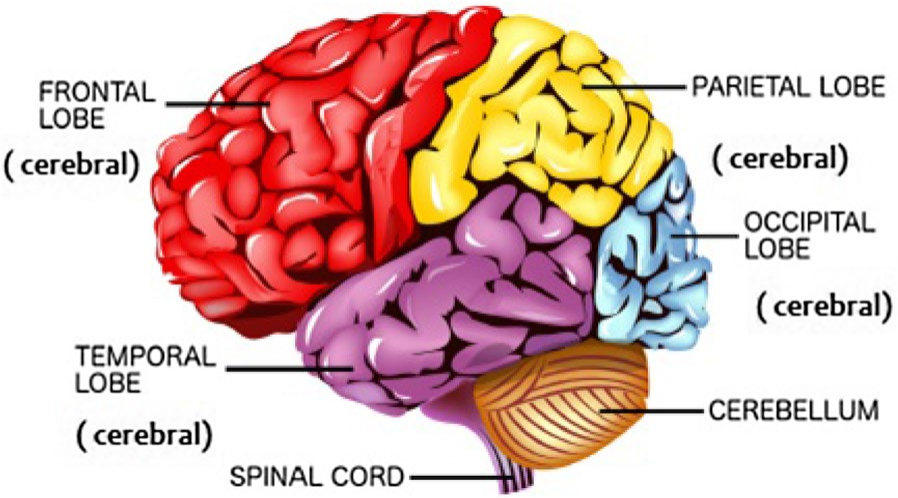
Functional diagram of brain diagram is adopted from [5]

## Functional structure of the brain

Before understanding EEG signals, we need to understand the structure of the brain. The human brain conveyed into three critical parts: cerebrum, cerebellum and cerebrum stem. Cerebrum subdivided into frontal lobe, parietal lobe, temporal lobe, occipital lobe, insular and limbic lobe alludes Fig. 1. Each part connected with some mental capacity, for example, the parietal projection sees agony and taste sensations and is associated with critical thinking exercises. The temporal lobe worried about hearing and memory. The occipital lobe primarily contains the districts utilised for vision-related errands. The frontal lobe principally connected with feelings, critical thinking, discourse and movement [6, 7]. A grown-up human brain holds, on an average, 100 billion neurons [8]. Neurons process and send data through electrical and chemical signals due to this it generates neuronal oscillations called brainwaves or EEG signals. Table 1 shows electrical and functional characteristics of these waves. The frequency range of EEG signals is 0.5–100 Hz, whereas amplitude range is 10–100 μV [9]. Delta wave has highest amplitude and lowest frequency, whereas gamma waves have highest frequency and lowest amplitude. In reviews, the frequency range varies by ± 0.5–1 Hz.

**Table 1:** Electrical characteristics of significant brainwaves

Brainwave	Frequency range (Hz)	Amplitude (μV)	Mental function
$\delta$	0–4	10–100	Unconsciousness during a deep dreamless sleep
			During a deep dreamless sleep
$\theta$	4–8	10–50	Subconscious mind
			Focused attention
			Emotion responses
$\alpha$	8–12	5–25	Relaxed mental state
$\beta 1$	12–16	0.1–1	Intense focused mental activity
$\beta 2$	16–30	< 0.1	Anxious alert
$\gamma$	30–99	≪ 0.1	Hyper brain activity

Amplitude values measured during data collections

**Fig. 2:**
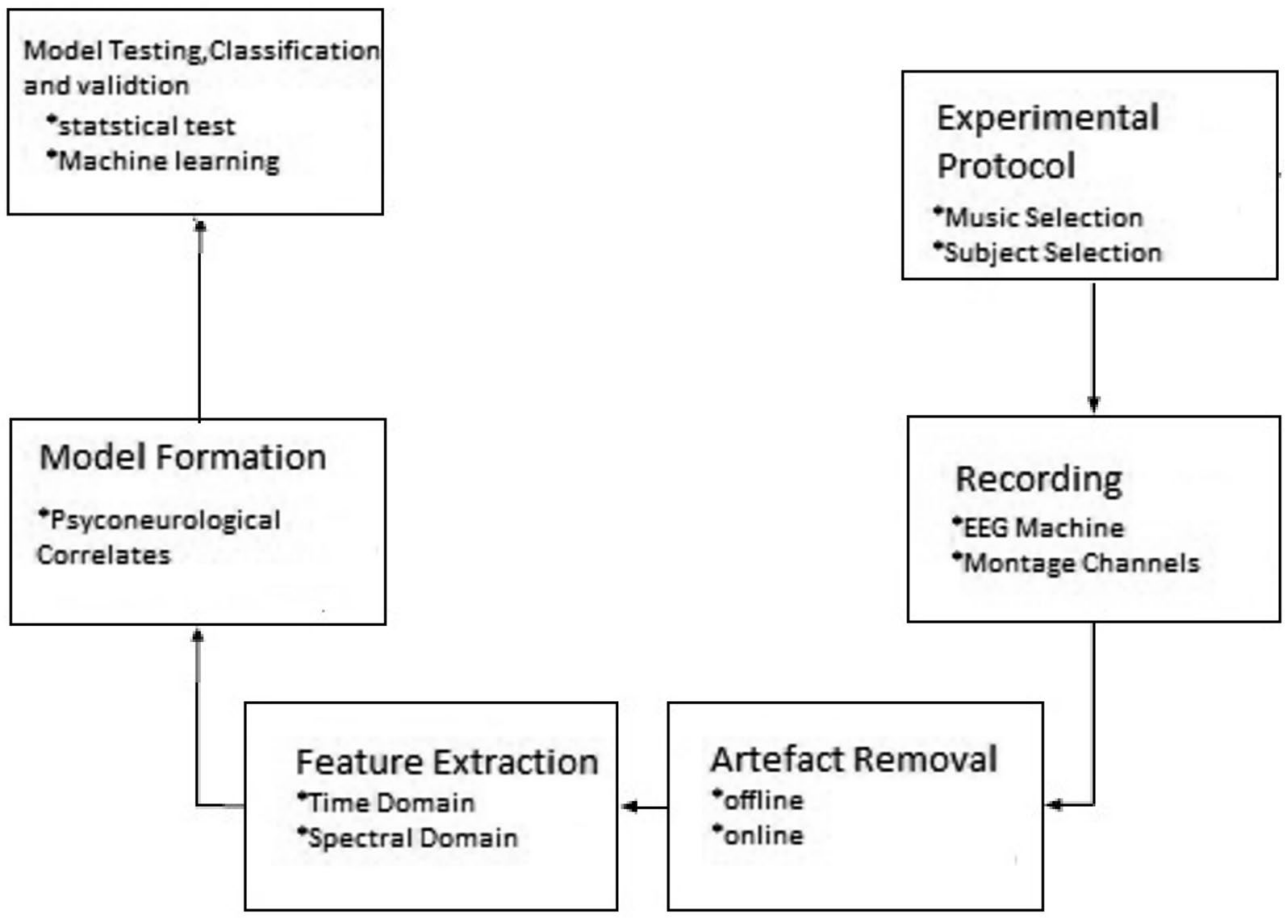
Experimental approach adopted in reviews

## Literature selection and analysis

The keywords used to select the article were EEG and Music and Emotions on a repository like PubMed, IEEE explorer, Science Direct and Mendeley research tool. Library recognised quality twenty-two papers from the year of 2001 and 2018 created using Mendeley [10]. The mostly followed research methodology is shown in Fig. 2. The articles were analysed concerning general steps observed in an experiment such as participants, stimulus, EEG machine, channel, montages preprocessing, feature extraction, statistical testing and machine learning.

## Summary of reviews

This section summaries findings and outcomes all the selected articles.

For a musical stimulus which was known to fluctuate in full of affective valence (positive versus negative) and intensity (extreme versus quiet), the author found that the pattern of asymmetrical frontal EEG activity. A higher relative left frontal EEG movement to satisfaction and cheerful melodic entries and more prominent relative right frontal EEG action to fear and dismal melodic selections. The author additionally discovered EEG asymmetry distinguished the intensity of emotion [11]. For the distinctive stimuli excerpt, jazz, rock-pop, traditional music and environmental sound. Author discovered positive enthusiastic attributions were joined by an expansion in left fleeting initiation, negative by a more two-sided design with predominance of the privilege fronto-temporal cortex. Author additionally discovered female members affirmed more prominent valence-related contrasts than males [12]. In this research, wonderful and offensive feelings were evoked by consonant and cacophonous melodic portion creator discovered lovely music was related with increment in frontal mid-line $\theta$ power [13]. In this examination, EEG-based emotion classification algorithm was explored utilising four types musical excerpts. The hemispheric asymmetry $\alpha$ power indices of brain activation were extracted as feature [14]. The author examined the connection between EEG signs and music-initiated emotion responses using four emotional music excerpts (Oscar film track). The author found that low-frequency bands $\delta$, $\theta$ and $\alpha$ are correlate of evoked emotions [15]. In this examination the author researches spatial and spectral pattern for evoked feelings because of melodic passage. Author found that spatial and spectral pattern most significant to feeling and reproducible crosswise over subjects [16]. In this investigation, the author distinguished 30 subject-free features that were most connected with emotion processing crosswise over subjects and investigated the convenience of utilising less electrode to describe the EEG flow amid music listening [17]. For stimulus rock-pop, electronic, jazz and broadband noise author examined the relation between subjects’ EEG responses to self-evaluated enjoyed or loathed music. Movement in $\beta$ and $\gamma$ band may prompt a relationship between music inclination and enthusiastic excitement phenomena [18]. In this article, author found frequency band, beta and theta, perform superior to anything other frequency band [19]. The author investigated like and disliked under three cases familiarity of the music by taking three types music regardless of familiarity of music, familiar music and unfamiliar music. The author found that familiar music gives highest classification accuracy compared to regardless familiarity and unfamiliar music [20]. The authors found that among the musician and non-musician subjects participated in the research, musicians have significantly lower frontal $\gamma$ activity during music listening and music imaging than resting state [21]. Author classified euphoric versus non-partisan, upbeat versus melancholic and well-known versus new melodic selection. The author researched brain network related to happy, melancholic and unbiased music. The authors research inter/intra provincial network designs with the self-announced assessment of the melodic selection [22]. The author found that among members thirty people of three distinctive age gatherings (15–25 years, 26–35 years and 36–50 years). The brain signals of age gathering (26–35 years) gave the best emotion acknowledgement accuracy in understanding to the self-reported emotions [23]. Author proposed a novel user identification framework using EEG signals while tuning in to music [24]. Authors quantify emotional arousal corresponding to different musical clips [25]. Author suggests that unfamiliar songs are most appropriate for the construction of an emotion recognition system [26]. The author explores the impact of Indian instrumental music Raag Bhairavi using frontal theta asymmetry [27]. The author proposes the frontal theta asymmetry model for estimating valence of evoked emotions and also suggested electrode reduction for neuromarketing applications [28, 29]. Author proposes frontal theta as biomarker of depression [30].

### Participants and their handedness

#### Handedness

Human brain has two identical anatomical spheres, but each sphere has functional specialisation. Handedness is concept which by simplistic definition is prominent hand used in day-to-day activity [31]. Each hemisphere has specific prominent function, like language abilities in left hemisphere in right-handed person [32]. As we are probably aware that the brain is cross-wired, the left side of the hemisphere of the cerebrum controls the right side of the body and vice versa in the majority of people. In research involving brain and stimuli, we first need to know about handedness as it is an indicator of prominent hemisphere. As a prominent hemisphere has specialised functions; observations, findings, interpretation differ according to dominance. Many functions change hemisphere as per dominance in particular person. Like, left-handed people have language processing in right hemisphere and right-handed have in left hemisphere [33]. Brain pattern of right- and left-handed persons are different [34]. This section analyses the natures of subjects considered in the reviews

**Table 2:** Analysis on the basis of participant and handedness

References	Participant	Handedness inventory
[11]	59 (29 males, 30 females) right-handed	Edinburgh
[12]	16/right-handed	Edinburgh
[13]	22 non-musicians	Edinburgh
[14]	5 Normal	NA
[15]	26 Normal	NA
[16]	26 Normal	NA
[17]	26 Normal	NA
[35]	79 depressed	NA
[18]	9 right-handed normal	NA
[19]	5 right-handed normal	NA
[21]	6 Musicians (4 men and 2 women)	NA
	5 healthy non-musicians (4 men and 1 woman).	
[20]	9 right-handed normal	NA
[36]	13 right-handed normal	NA
[22]	19/non-musicians (11 females and 8 males)	NA
[23]	30/men and women of three different age groups	NA
	(15–25 years, 26–35 years and 36–50 years)	
[24]	60 Normal	NA
[25]	5/(M = 3, F = 2)	NA
[26]	15 normal	NA
[27–29]	41 normal right-handed	Edinburgh
[30]	23 depressed 17 normal right-handed	Edinburgh

*NA* not available

#### Participants

Subjects used 5–79 with median 20 most of the researchers consider unbalanced numbers of males and females see Table 2. When subjects participated in studies are less, outcome of the hypothesis is always questionable. In 78% of research, authors reported right-handed subjects without any handedness inventory. Only 22% of research used handedness Edinburgh inventory [37, 38]. In most of the investigation, 95% researcher recruited normal participants; few of them verify the normalcy. Most of the researchers selected participants who are the students or working staff and of the same background. Author [23] investigated the impact of the musical stimulus on a different age group. Author [21] studied the effect of the musical stimulus by recruiting musician and non-musician subject. Authors [30, 35] investigated the impact of the musical stimulus on mentally depressed subjects.

### Musical stimulus type, duration and emotions

**Table 3:** Analysis on the basis of stimulus and emotions

Reference	Stimulus duration/type	Emotions
[11]	60 s/excerpts vary in affective valence	Fear, Joy
	and intensity (i.e. intense vs. calm)	Happy, Sad
[12]	15 s/Jazz, rock-pop, classical music	Positive, Negative
	and environmental sounds	
[13]	1 min/Consonant comprised 10 excerpts of joyful instrumental dance tunes	Pleasant, Unpleasant
	Dissonant stimuli were electronically manipulated counterparts of the consonant excerpts:	
[14–17]	30 s/Four types musical excerpt	Joy, Angry,
		Sadness, Pleasure
[35]	5 min/West-African Djembe drums and electronic hand drums	Depression
[18]	15 s/Rock-pop, electronic, jazz and classical (15 excerpts per genre) and 15 excerpts of broadband noise	Like, Dislike
[19]	3 min/16 peace of music	Exciting, Relaxing
[21]	2.5 min/Largo, D-flat major, Going Home	NA
[20]	60 musical excerpts LD (regardless of familiarity), LDF (familiar music), LDUF (Unfamiliar music))	NA
[36]	15 s/10 film music excerpts	Anger, Fear, Happiness,
		Sadness, Tiredness
[22]	60 s/Iranian music along with other classical excerpt	Valence, Arousal
[23]	1 min /Rap, metal, rock and hip-hop genres Happy	
[24]	20 s/Electronic, classical and rock. four music genres	Anger, Happiness, Calm, Sadness, Scare
[25]	30 s/8 cross-culture instrument	Joy, Sorrow, Anxiety, Calm
[26]	2 s/Familiar unfamiliar musical stimuli	Like, Dislike
[27–29]	10 min/Instrumental Raag Bhairavi	Like, Dislike
[30]	10 min/Instrumental Raag Bhairavi	Like, Dislike, Depression

Different genres of musical stimulus excerpt of pleasant and unpleasant music selected to evoke a different types emotions stimulus chosen are classical, rock, hip-hop, jazz, metal, African drums, Oscar tracks, environmental (refer Table 3). Author [13, 18] used noise along with pleasant stimulus to elicit negative emotion. Authors [20] used familiar unfamiliar and regardless familiar music. Stimulus duration selected from 2 s to 10 min with median 30 s. Different excerpts interleaved with some time gap. Self-responses of evoked emotion noted from subjects participated in study. Emotions investigated the positive and negative emotions such as Fear, Happiness, Sadness, Anger, Tiredness, Like, Dislike, Anxiety, and Depression. Some authors used feel tracer to measure arousal effect of the stimulus.

### EEG machine and channel investigated

**Fig. 3:**
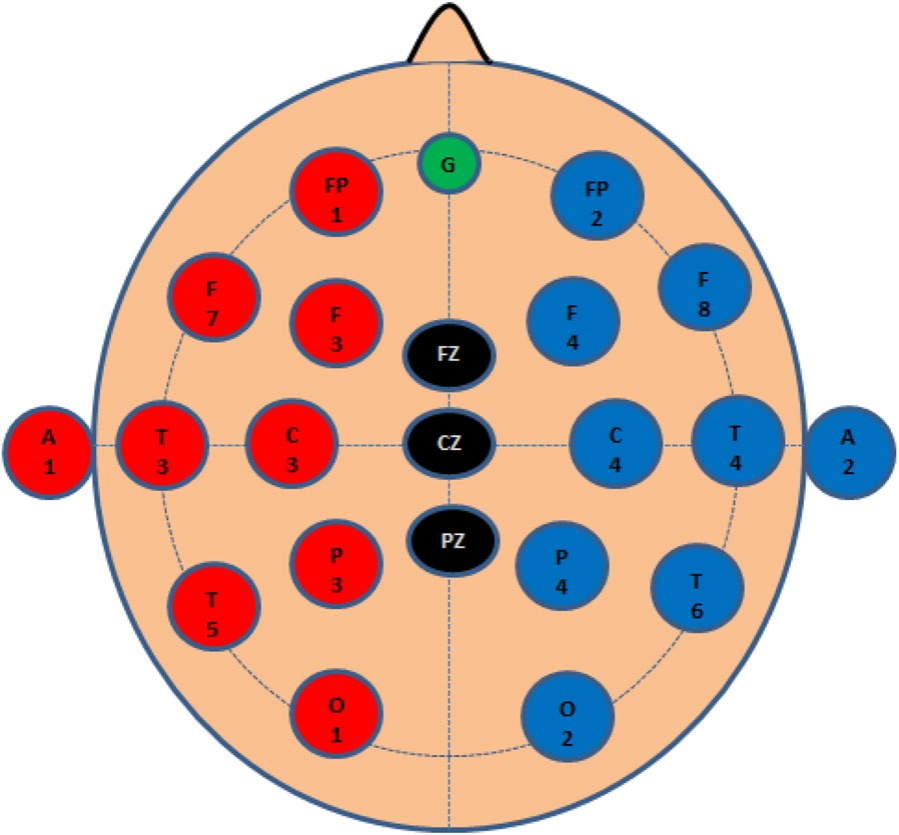
10–20 System of electrode placement

Twelve different EEG machines are used in the reviewed articles (refer Tables 4 and 5). All the EEG machines surveyed on the features Compliance Certification, PC Interface, Filter Number of the channel, Sampling Frequency, Compatible toolbox, Electrode Type. Almost all machines were FDA (Food and Drug Administration), CE (Conformite Europeenne) certified, required a sampling frequency. Most of the equipment and compatible toolbox MS-Excel/MATLAB/LabVIEW. Mostly, 10–20 systems of electrode placement used in reviews (refer Fig. 3). Electrode is used in the reviewed articles 1–63 with a median 21.5. Total of 75% article reported referential montages taking A1 and A2 reference electrodes. Author [11, 35] used vertex electrode Cz as reference. Author [20] used frontal mid-line electrode Fz as well as A1 and A2 reference electrodes. Author [18] used Laplacian montage.

**Table 4:** EEG machine and sampling frequency

References	EEG machine	Sampling frequency (Hz)
[11]	Electro-Cap, Inc.	512
[12]	Electro-Cap International, Eaton OH	100
[13]	Electro-Cap International	500
	Inc., Eaton, USA	
[14–17]	NeuroScan Inc.	500
[35]	Bio Semi Active II amplifier	2048
[18]	g.MOBIlab	256
[19]	ESI NeuroScan	500
[21]	Elekta-Neuromag	NA
[20]	g.MOBIlab	256
[36]	Biosem	512
[22]	Electro-Cap International	128
	Inc., Eaton, USA/1	
[23]	Emotiv	256
[24]	Neuro-headset Emotiv	128
[25]	Recorders and Medicare	256
	Systems	
[26]	Waveguard cap	256
[27–29]	Neuromax 32 Medicaid	256
[30]	Neuromax 32 Medicaid	256

**Table 5:** Channels and Montages

Reference	Channel investigated	Montage
[11]	F3, F4, P3 and P4	Referential Cz
[12]	Fp1, Fpz, Fp2, F7, F3, Fz, F4 F8, FT7, Fc3, FC4, FT8, T7, C3, Cz, C4, T8, Tp7, Cp3Cp4, Tp8, P7, P3Pz, P4 P8, O1 and O2	Referential Ear
[13]	AF4, F4, F8, FC4 AF3, F3, F7, FC3; C3, C5, CP3, CP5 C4, C6, CP4, CP6; P3, P5, PO3, PO7. P4, P6, PO4, and PO8.	
[14–17]	Fp1-Fp2, F7-F8, F3-F4, FT7-FT8, FC3-FC4, T3-T4, T5-T6, C3-C4, TP7-TP8, CP3-CP4, P3-P4, O1-O2	Referential
[35]	Fp1-Fp2, F3-F4, F7-F8	Referential Cz
[18]	AF3, F7, F3, FC5, T7, P7, O1, O2, P8, T8, FC6, F4, F8, and AF4	Referential laplacian
[19]	Fp1, F7, F3, FT7, FC3, T7, P7, C3, TP7, CP3, P3, O1, AF3, F5, F7, FC5, FC1, C5, C1, CP5, CP1, P5, P1, PO7, and Fp2, F8, F4, FT8, FC4, T8, P8, C4, TP8, CP4, P4, O2, AF4, F6, F8, FC6, FC2, C6, C2, CP6, CP2, P6, P2, PO8, PO6, PO4, CB2	Referential
[21]	NA	Referential
[20]	AF3, F7, F3, FC5, T7, P7, O1, O2, P8, T8, FC6, F4, F8, and AF4	Referential laplacian
[36]	128 electrodes	Referential
[22]	AF3, F7, F3, FC5, T7,P7,O1, O2, P8, T8, FC6, F4, F8, AF4.	Referential
[23]	Fp1	NA
[24]	AF3, AF4, F3, F4, F7, F8, FC5, FC6, P7, P8, T7, T8, O1, O2	
[25]	NA	Referential Fz
[26]	Fp1, Fp2, F3, F4, F7, F8, Fz, 3, C4, T3, T4, and Pz	Referential Cz
[27–29]	FP1, F7, F3, FP2, F8, F4	Referential
[30]	FP1, F7, F3, FP2, F8, F4	Referential

### Preprocessing for artefact and feature extraction

Most of the articles reported manual, and offline removal artefact; few articles used filter and Laplacian montage method [19]. The notch filter was also used to remove features extraction transform. Most of the articles used FFT either DFT or STFT (56.25$\%$); 12$\%$ researchers used wavelet transform and 6.25 $\%$ researcher applied DFA and time domain analysis. Author [18] applied time–frequency transform (Zhao-Atlas-Marks, STFT, Hilbert, Huang Spectrum) (refer Table 6).

### Brainwave and location investigated and statistical test

**Table 6:** Analysis on the basis preprocessing for artefact removal and feature extraction transform

Reference	Preprocessing approach	Feature extraction
[11]	Offline manual	FFT
[12]	Offline manual	Time domain
[13]	Offline manual	FFT
[14]	Filter of 0–100 Hz, notch filter of 60 Hz and offline manual	STFT
[15]	Filter of 0–100 Hz, notch filter of 60 Hz and offline manual	STFT
[16]	Filter of 0–100 Hz, notch filter of 60 Hz and offline manual	STFT
[17]	Filter of 0–100 Hz, notch filter of 60 Hz and offline manual	STFT
[35]	Offline manual	FFT
[18]	Offline manual	Time-frequency transform (Zhao-Atlas-Marks STFT, Hilbert Huang Spectrum)
[19]	Offline manual	STFT
[21]	Offline manual	Wavelet
[20]	Offline manual	TF
[36]	Filter, offline manual, PCA	FBCSP
[22]	Offline manual	DTF
[23]	Offline manual	hybrid domain
[24]	Offline manual	Wavelet
[25]	Offline manual	DFA
[26]	EEG Lab Tool, ICA	FFT
[27–29]	Instrumental Raag Bhairavi	FFT
[30]	Instrumental Raag Bhairavi	FFT

*FFT* Fast Fourier transform, *STFT* short Fourier transform, *TF* time frequency, *DFA* dendred facture analysis, *ICA* independent component analysis, *FBCSP* filter-bank common spatial patterns

31.25$\%$ researchers investigated all brainwaves ($\delta$, $\theta$, $\alpha$, $\beta$ and $\gamma$) together. Remaining of them selected few of them or independently studied a single band. In all reviews $\alpha =75\%$, $\gamma=37.5\%$, $\beta =56.25\%$, $\delta =37.5\%$ and $\theta=87.5\%$ were investigated. Almost all researchers investigated frontal hemisphere only. Author [20] investigates all regions of the brain and correlates $\gamma$ waves with memory processing. Twenty five per cent reviews conducted statistical tests, namely ANOVA, *t* test and Z test. Most of the authors consider confidence level of 0.05. Seventy-five per cent reviews directly applied machine learning algorithm (refer Table 7).

**Table 7:** Brainwave, location investigated and statistical test

Reference	No. of band /Brainwave/Location investigated/Brain model	Statistical test
[11]	2/$\alpha, \theta$/Frontal/Asymmetry	ANOVA
[12]	-/-/Frontal/Asymmetry	ANOVA
[13]	4/other waves and $\theta$/Frontal/Asymmetry	ANOVA, paired *t* test
[14]	1/$\alpha$/Entire/Asymmetry	NA
[15]	5/$\delta$,$\theta, \alpha$, $\beta$,$\gamma$ /Entire/Asymmetry	NA
[16]	5/$\delta$,$\theta, \alpha$, $\beta$,$\gamma$ /Entire/Asymmetry	NA
[17]	5/$\delta$,$\theta, \alpha$, $\beta$,$\gamma$ /Entire/Asymmetry	NA
[35]	2/$\theta, \alpha$ /Frontal/Asymmetry	*z* test
[18]	4/$\theta ,\alpha$, $\beta$,$\gamma$	NA
[19]	5/$\delta$,$\theta ,\alpha$, $\beta$,$\gamma$ /Entire/Asymmetry	NA
[21]	5/$\delta$,$\theta ,\alpha$ ,$\beta$,$\gamma$ /Entire/Asymmetry	*z* test
[20]	5/$\delta$,$\theta ,\alpha$ ,$\beta$,$\gamma$ /Entire/Asymmetry	NA
[36]	5/$\delta$,$\theta ,\alpha$ ,$\beta$,$\gamma$/Entire	NA
[22]	4/$\theta ,\alpha$ ,$\beta$,$\gamma$ /Entire/Asymmetry	NA
[23]	NA	NA
[24]	4/$\theta ,\alpha$ ,$\beta$,$\gamma$ NA	
[25]	3/$\alpha$ ,$\beta$,$\gamma$	NA
[26]	5/$\delta$,$\theta ,\alpha$ ,$\beta$,$\gamma$	ANOVA
[27–29]	1/$\theta$/Frontal	*t* test
[30]	1/$\theta$/Frontal	*t* test

ANOVA—Analysis of Variance

### Machine learning algorithms

In all, 72%$\%$ reviews employed supervised learning algorithm, namely k-NN, SVM, MLP, LDA, QDA, HMM, self-responses of subjects used as a feature vector. Twenty-eight per cent$\%$ reviews used statistical tests, namely *t* test, ANOVA and *Z* test. Forty per cent$\%$ of reviews used SVM along with other classifiers for classifying emotions. Classification accuracy is the most used metric. No study reported unsupervised machine learning algorithms (see Table 8).

**Table 8:** Machine learning algorithms and model evaluation attributes

Reference	Machine learning algorithm	Model evaluation attributes
[11]	NA	*p* value
[12]	NA	*p* value
[13]	NA	*p* value
[14]	MLP	CA
[15]	SVM	CA
[16]	SVM	CA
[17]	SVM, MLP	NA
[35]	NA	NA
[18]	SVM, QDA, k-NN	NA
[19]	k-NN, SVM	NA
[21]	NA	*p* value
[20]	k-NN, SVM	NA
[36]	NA	NA
[22]	SVM	NA
[23]	K-nn, SVM and MLP	CA
[24]	SVM, HMM	CA
[25]	NA	NA
[26]	NA	*p* value
[27, 28]	NA	*p* value
[29]	k-NN,LDA	CA
[30]	NA	*p* value

*CA* classification accuracy, *MLP* multi-level perception, *SVM* support vector machine, *k-NN* K-nearest neighbour, *LDA* linear discriminant analysis, *QDA* quadratic discriminant analysis, *HMM* hidden Markov level

## Discussion and recommendations

### Participants

The vast majority of the engineering domain study consider very less subject on an average 11 approximately, especially articles on IEEE explorer. To prove the hypothesis, minimum 30 subjects are required in the study [39]. In case scholars use subjects of both sexes, the number of subjects should be equal. Most of the authors required normal subjects without confirming normalcy of subjects. Homogeneous population were considered. This study is multidisciplinary study human factor, and experimental psychology is involved in this [40]. Most of the studies conducted by engineering fraternities are without clinical guidance. Handedness not considered if it considers evasive about handedness evaluation method.

### Musical stimulus and dimension of emotion

**Fig. 4:**
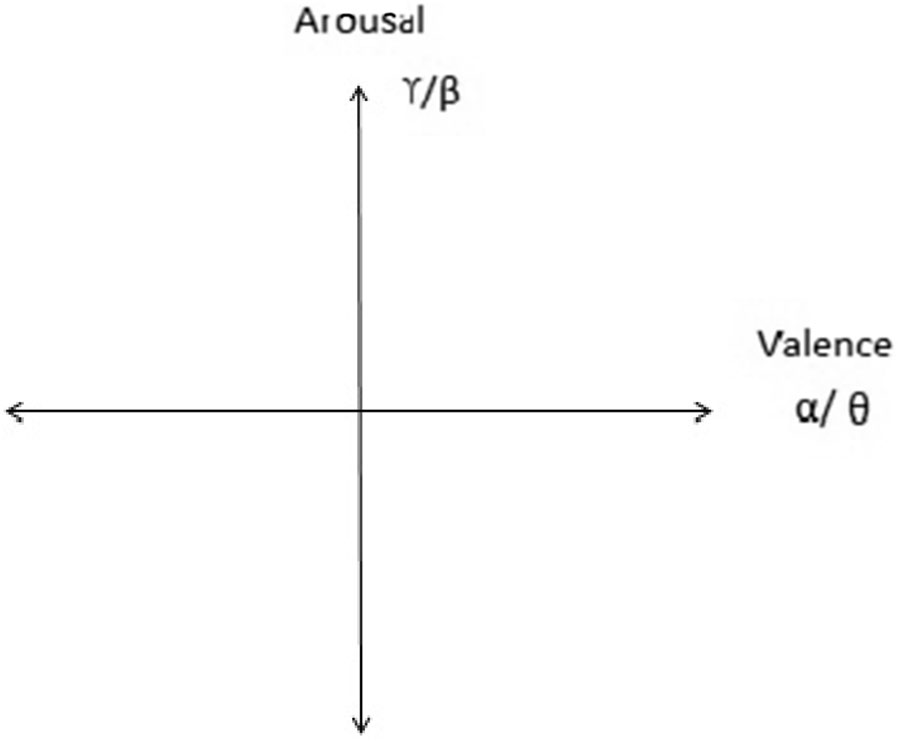
Recommended 2D model

Reviews use various genres of musical stimuli. To evoke different emotions among the subjects, a different emotional excerpt of incentives was employed. Most of the reviews employed familiar musical stimulus. Author [26] empirically proved unfamiliar excerpt most suitable for the construction of an emotion identification system. In reviews, various emotions are considered for emotion classification. The higher number of emotions causes emotion acknowledgement troublesome, and a few emotions may overlap [41]. In most surveys, the 1-dimensional emotion model was used. To investigate arousal feel tracer used feel tracer instrument is not reliable [42]. No reviews report about an automatic prediction of valence and arousal of 2 dimensional for the same excerpt of musical stimuli. High-frequency brainwaves like beta and gamma were used to correlate arousal [43] of emotion, while low frequency like alpha or theta for valence of emotion [11, 13]. Arousal and valence for the same excerpt of stimulus were plotted on the same graph as shown in Fig. 4.

#### Emotional processing in depression

Emotions are broadly classified as positive and negative, for sake of their understanding in processing in brain. Broadly, it is seen that positive emotions are processed in left anterior hemisphere (a.k.a. prefrontal cortex) of brain and negative emotions are processed in right [44]. In cases of depression, hypothesis in left anterior hemisphere hypo-arousal or right anterior hemisphere hyper-arousal leads to symptoms of depression [45]. EEG pattern supports evidence; findings shows that in cases of depression left anterior hemisphere is relatively inactive to right hemisphere [27], indicating that patients with depression have differential processing of stimuli than people without depression.

### EEG machine and montages

While selecting EEG machine, following features should be considered

- Minimum 256 Hz sampling frequency
- CE, FDA approvals
- Compatible with MS-EXCEL, LabVIEW
- Quick technical support
- DC operated

Montages are sensible and efficient game plans of electrode sets called channels that show EEG action over the whole scalp, permit appraisal of movement on the two sides of the cerebrum (lateralization) and aid in localisation of recorded activity to a specific brain region [46]

- Bipolar Montage

In a bipolar montage, each waveform signifies the difference between two adjacent electrodes. This class of montage is designated as longitudinal bipolar (LB) and transverse bipolar (TB). Longitudinal bipolar montages measure the activity between two electrodes placed longitudinally on scalp, e.g. Fp1-F7, Fp1-F3, C3-P3, Fp2-F8, Fp1-F3, Fp2-F4, Fp2-F8 and F3-C3. Transverse bipolar montage measures activity between two electrodes along crosswise, e.g. Fp1-Fp2, F7-F3, Fp1-Fp2, F7-F3, Fp2-F8, F3-Fz, Fp2-F8, F3-Fz and F7-F3

- Bipolar Referential Montage

In this montage, the distinction between the signal from an individual electrode and that of an assigned reference electrode was estimated. The reference electrode has no standard position. Nonetheless, the situation of the reference electrode is unique in relation to the account electrode. Mid-line positions are often used to avoid amplification of signals in one hemisphere relative to the other. Another most loved reference that utilised impressively is the ear (left ear for left hemisphere and right ear for right hemisphere), e.g. the left and right ears are considered as reference electrode Fp1-A1, Fp2-A2, F7-A1, F8-A2, Fp1-Cz, Fp2-Cz, F7-Cz, F8-Cz and so forth

- Laplacian Montage

In this montage, the distinction between a electrode and a weighted normal of the encompassing electrodes is utilised to represent a channel.

### Preprocessing for artefacts

EEG recording is exceedingly powerless to various forms and sources of noise. Morphology, an electrical characteristic of artefacts, can lead to significant difficulties in analysis and interpretation of EEG data. Table 9 shows various types of artefacts. The morphology of external artefacts is easily distinguishable from actual EEG [47]. Taking long duration and using many electrode artefact-free recording protocol is the best strategy for preventing and minimising all types of artefacts [27]

- Educate the members around an eye, physical movement
- Try not to permit electronic contraption in EEG recording lab
- Record in acoustic free, diminish light and at surrounding temperature
- All muscle, ocular or movement artefact slots of EEG signals reject
- Members wash their hair to expel oil from their scalp.
- Use proper montage

**Table 9:** Various artefacts in EEG signal recording

Category	Artefact /Source(Cause)/Frequency/Amplitude Morphology	Artefact Prevention
Physiological Artefacts	Cardiac/Heart/$\le$1Hz/1-10mV /Epilepsy	Selection of proper montage Monitoring during recording Offline visual inspection Low pass filter (LPF) Data Rejection
	EOG/Eye/0.5-3 Hz/100mV /Tumour, delta wave	Artefact-free recording protocol Online monitoring Offline visual inspection Low pass filter (LPF) Using various Transform (ICA, PCA, EOG subtraction)
	Muscle Artefact/Muscle /$\ge$ 100Hz/low /Beta frequency	Artefact-free recording protocol Online Monitoring Offline visual inspection High pass filter(HPF) Data rejection
	Physical movement artefact /Physical movement /Very low/ very high /Morphology different from actual EEG	Artefact-free recording protocol Online monitoring Offline visual inspection Data rejection
External Artefacts	Transmission line /Transmission line 50–60 Hz/low/ Morphology different from actual EEG	Notch filter DC power supply
	Phone artefacts /Mobile and landline phone /high/high/different	Artefact-free recording protocol
	Electrode artefact /Electrode and sweating /very low/high	Artefact-free recording protocol LPF
	Impedance artefact /Electrode with impedance>5K$\Omega$ /-/approximately 100 μV/different	

### Feature extraction

There are three methods of analysing EEG signal time domain, frequency domain, time–frequency domain [9]

- Time domain

All real-world signals presented time domain. This method is suitable to visualise real-world signal, voltage, PSD (power spectral density) and energy estimation of signal, mostly used for epilepsy analysis.

- Frequency domain

Analysis of EEG signals concerns frequency, rather than time. It gives PSD’s of various rhythms of EEG signals. It is suitable for studying various brainwaves over a stipulated time period

- Time frequency

Time–frequency examination contains those procedures that review a signal in both the time and frequency at the same time, appropriate for event-related emotion acknowledgement.

### Brainwave and location

In existing literature, a frontal region mostly explored as it associated with emotion processing. A few researchers investigated an exclusive wave correlating evoked emotion. As mentioned in Sect. 1, musical stimulus created many psychological changes in subjects only examining frontal region, and few are the wave is not enough in creating a model of evoked emotion. Various lobes and many waves establishing their interrelationship need to be explored.

### Machine learning algorithm

SVM is a supervised machine learning algorithm which can be used for classification or regression problems. It is a suitable algorithm for classification of evoked emotions. SVM utilises kernel trick to transform the data, and after that, because of these changes, it finds an ideal limit between the conceivable yields. Nonlinear kernel tricks can catch substantially more perplexing connections between data points without having to perform difficult transformations on own [48]. It has features

- High prediction speed
- Fast training speed
- High accuracy
- Results are interpretable
- Performs wells with small numbers of observation

### Model performance metrics

Healthcare and engineering models have different obligations, so the assessment metric should be different and should not be judged using a single metric; classification accuracy metrics are mostly considered in reviews for assessing the model. The model performance represented in the form of the confusion matrix is shown in Eq. (1).

1 $$\begin{aligned} {\hbox {Cp}} \left[ \begin{array}{ll} {\hbox {Tp}} &{} {\hbox {Fp}} \\ {\hbox {Fn}}&{} {\hbox {Tn}} \end{array}\right] \end{aligned}$$

2 $$\begin{aligned} {\hbox {Accuracy}}&=\frac{{\hbox {Tp}}+{\hbox {Tn}}}{{\hbox {Tp}}+{\hbox {Tn}}+{\hbox {Fp}}+{\hbox {Fn}}} \end{aligned}$$

3 $$\begin{aligned} {\hbox {Cp}} \left[ \begin{array}{ll} 0&{} 0 \\ 25 &{} 125 \end{array}\right] \end{aligned}$$

4 $$\begin{aligned} {\hbox {Accuracy}}&=\frac{0+125}{0+125+0+25} \end{aligned}$$

Assume the inadequate model shown by Eq. (3) is having true-positive and false-positive values zero; still model classification accuracy by Eq. (2) is 83.33%. Accuracy is not a reliable metric for assessment of model. Apart from classification accuracy, there are many metrics for models assessment such as sensitivity, specificity, precision NPV (negative prediction value), FDR (false discovery rate), F1 score, FPR (false-positive rate), FNR (false-negative rate) accuracy, MCC (Mathew correlation coefficient) informedness (Youden index), markedness and ROC (receiver output character). Model performance metric such as recall, specificity, precision and accuracy are biased metrics [49]. ROC diagrams depicted the trade-off inside hit rates and false alert rates of classifiers and honed for the long time [50, 51]. As ROC decouples models performance from class skew and error costs, this makes ROC best measure of classification performance. The ROC graphs are useful for building the model and formulating their performance [52]. For a small number of positive class, F1 and ROC give a precise assessment of models [53, 54].

## Suggested approach

**Fig. 5:**
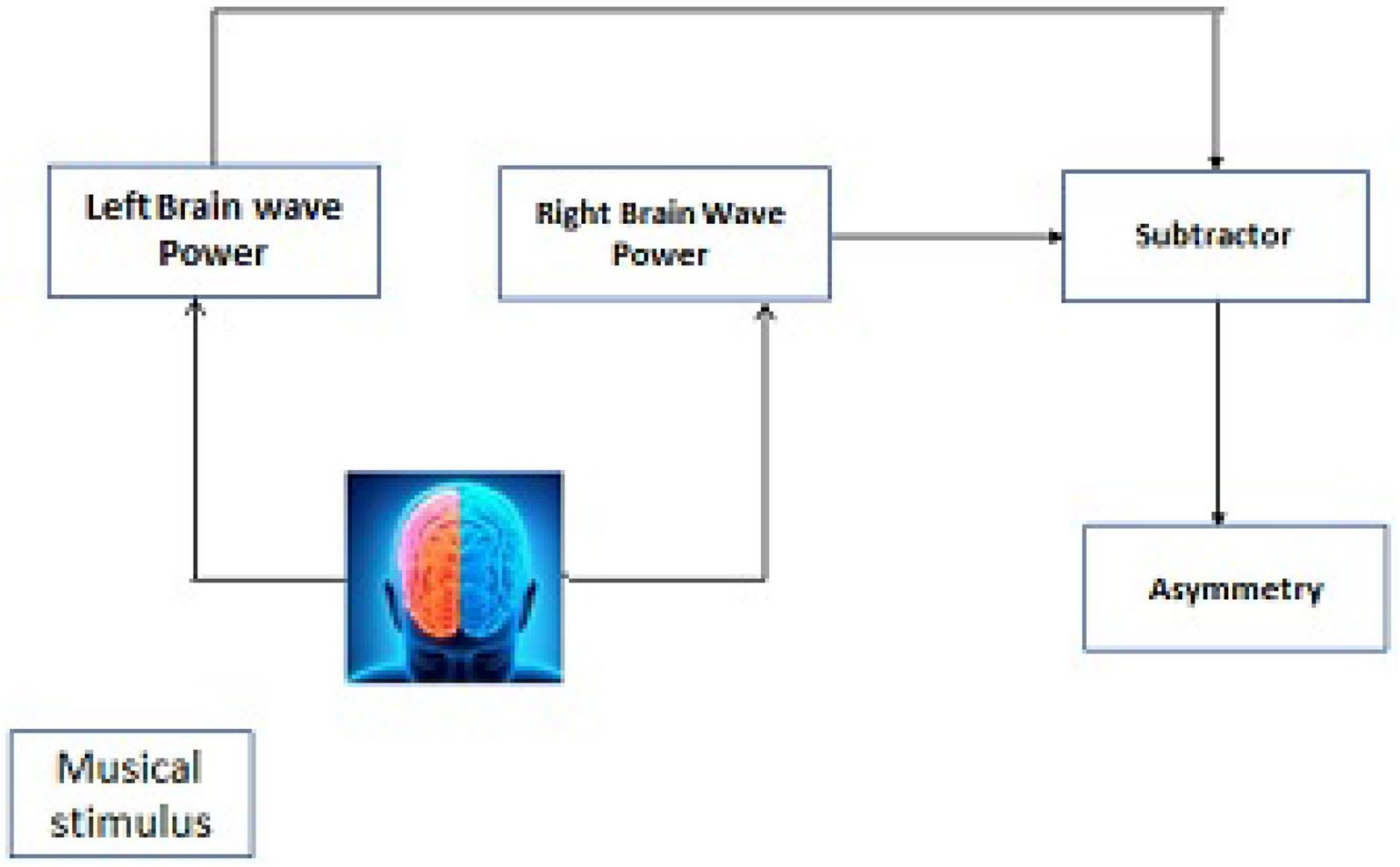
Asymmetry model

**Fig. 6:**
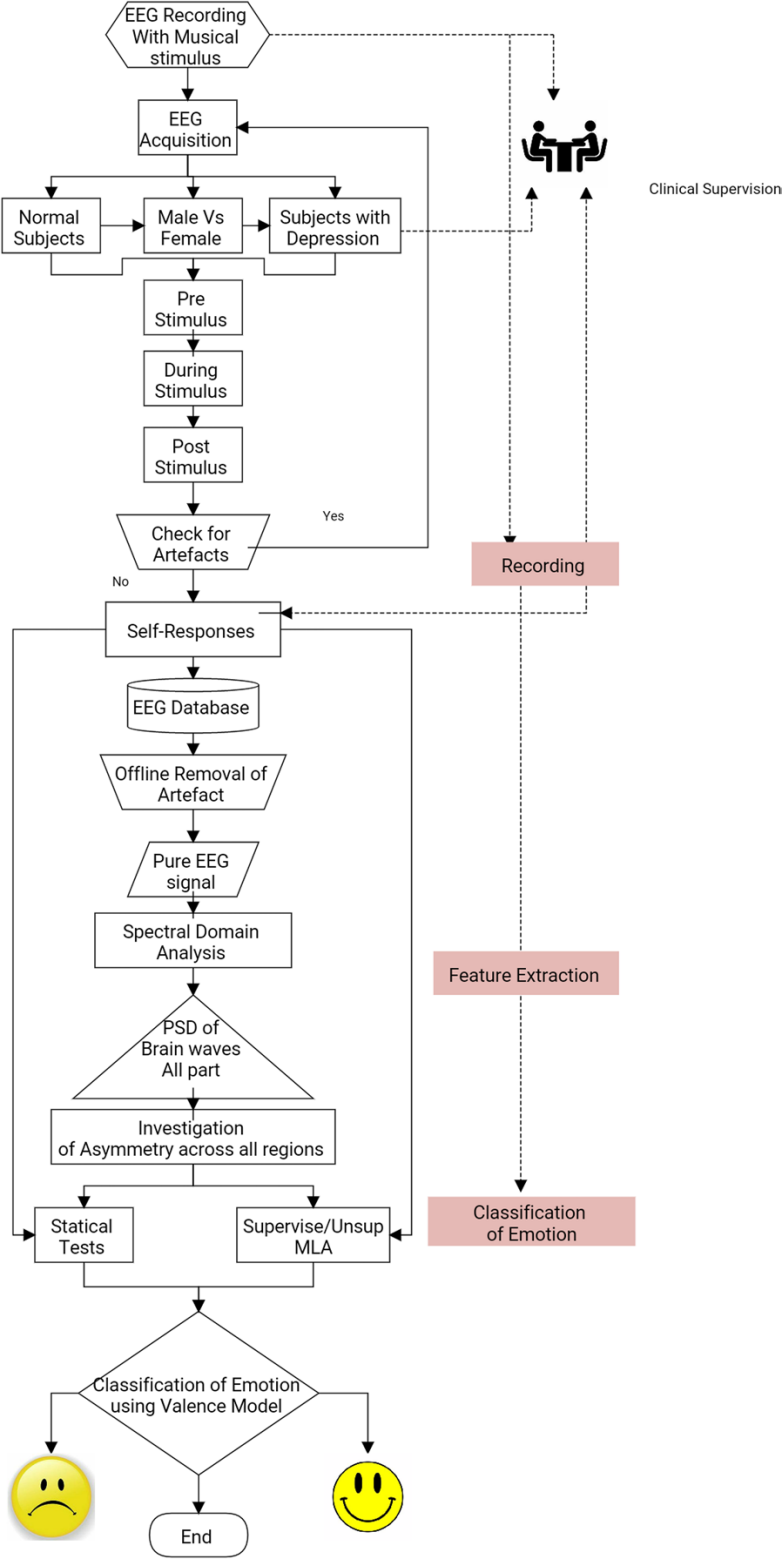
Suggested approach

As this research is interdisciplinary collaborative research by involving the medical fraternity of psychiatry or neurology background, music expert will satisfy Brouwer’s [40] recommendation I, II and VI. By recording EEG in three continuous gatherings, prestimulus, during stimulus and post-stimulus, could help in comparing with the baseline changes, and post hoc selection of data satisfy Brouwer’s recommendation III. moreover, remaining Burrowers recommendation IV and V by recording EEG using good artefact removing the protocol mentioned in Sect. 4.4 and Table 9. Analysing data using proper statistical test and machine learning algorithms (refer Fig. 6 for suggested approach). Comparison of left and right hemispheric activity refer gives vivid results, and the model formed called asymmetry model (refer Fig. 5). Most of the reviews compared left brain activity with the right brain activity and found that mathematical relationship for stimulus will be more significant.

## Conclusion

We have summarised, analysed and discussed the research articles with using keywords music, EEG and emotion from the year 2001-2018. We have outlined attention of different approaches considered in mental and emotion detection with the musical stimulus, We have drawn attention to various aspects of current research such emotion model, statistical test and machine learning algorithms, model performance metrics, etc. We have recommended best practices for putting scholar before the researcher. It will provide inputs for the new researcher in this area.
